# Effectiveness analysis of resistance and tolerance to infection

**DOI:** 10.1186/1297-9686-43-9

**Published:** 2011-03-01

**Authors:** Johann C Detilleux

**Affiliations:** 1 Quantitative Genetics Group, Faculty of Veterinary Medicine, University of Liège, Liège, Belgium

## Abstract

**Background:**

Tolerance and resistance provide animals with two distinct strategies to fight infectious pathogens and may exhibit different evolutionary dynamics. However, few studies have investigated these mechanisms in the case of animal diseases under commercial constraints.

**Methods:**

The paper proposes a method to simultaneously describe (1) the dynamics of transmission of a contagious pathogen between animals, (2) the growth and death of the pathogen within infected hosts and (3) the effects on their performances. The effectiveness of increasing individual levels of tolerance and resistance is evaluated by the number of infected animals and the performance at the population level.

**Results:**

The model is applied to a particular set of parameters and different combinations of values. Given these imputed values, it is shown that higher levels of individual tolerance should be more effective than increased levels of resistance in commercial populations. As a practical example, a method is proposed to measure levels of animal tolerance to bovine mastitis.

**Conclusions:**

The model provides a general framework and some tools to maximize health and performances of a population under infection. Limits and assumptions of the model are clearly identified so it can be improved for different epidemiological settings.

## Background

The breeding objective in most livestock species is to increase profit by improving performance efficiency. One way to reach this objective is to improve the animals' health, for example, through the implementation of appropriate management methods (e.g. chemotherapy, vaccination, and control of disease vectors). A more sustainable method consists in taking advantage, by selective breeding, of the within-breed variation that exists in the mechanisms of defenses against infectious pathogens [1]. Indeed, hosts have evolved resistance and tolerance defenses [2], thus breeders may choose, as progenitors, animals with the highest levels of resistance, tolerance, or both. One the one hand, resistance is the ability of the host to reduce the success of infection or to increase the rate of clearance of the pathogens. On the other hand, tolerance is the ability to reduce the detrimental effects of the pathogens on the performances of the hosts, either directly or by limiting immunopathological mechanisms [3]. The rate of transmission diminishes naturally among resistant hosts but not necessarily among tolerant ones, as these harbor the pathogen with no or moderate loss in performance [4].

Resistance and tolerance are associated with fitness costs, which arise from the diversion of limiting resources away from biological processes related to performance [5]. If these costs are too high, they may outweigh the effectiveness of the chosen strategy. Direct evidence of such costs can be found in experiments in insects [6], rainbow trout [7], crustaceans [8], wild birds [9] and mice [10].

To decide whether improving resistance, tolerance, neither, or both is the most effective strategy, it is proposed to (1) characterize the dynamics of the pathogens within and between hosts in the population under study, (2) evaluate the impact of the infection on the performances of the population, and (3) choose the most effective strategy. The goal of this study is to illustrate the methodology with a non-lethal micro-parasitic disease in a population where hosts have different levels of resistance to multiplication of the pathogen and different levels of tolerance to damages induced directly by the pathogens.

## Methods

### Pathogen dynamics

The model chosen here to depict the dynamics of transmission of the infection in a herd is a stochastic version of the SIS (S for susceptible, I for infected) model for the spread of a disease in a closed population of N individuals [11]. This model is appropriate for infections with no permanent immunity after recovery, i.e. individuals are susceptible to the infection, potentially get infected, may recover and become susceptible again. The time-scale of the disease process is assumed to be short compared to the life length of the host and no demographic turnover (natural birth or death) is considered. The area occupied by parasites and hosts is constant, so that numbers and densities coincide. There is only a single non-evolving pathogen species within infected hosts. Once infected, hosts are immediately able to infect other individuals (no latent period). Within the host, the number of pathogens increases following a sigmoidal growth curve and is directly related to the number of immune constituents of the host response to the pathogen, with no distinction between innate and specific immunity. Recovered hosts are as susceptible to infection as naïve hosts and re-exposure does not accelerate development of the disease.

In mathematical terms, the process is described by a continuous time Markov chain, {C^i^_t_; t = 0 to T, i = 1 to N}, where C^i^_t _denotes the number of pathogens in the i^th ^host at time t. Units of time are chosen arbitrarily. The chain has three transition probabilities (over a small time interval Δt) reflecting the three events, i.e. invasion of a new host by the pathogen, its multiplication and its killing by the immune response of the host.

The first transition probability is the probability the i^th ^susceptible host is infected by C_min _pathogens:(1)

where o(Δt) tends to 0 when Δt is small, C_min _is the minimum number of pathogens necessary to have infection, β is the per-capita rate of successful transmission of C_min _pathogens from an infectious host to a susceptible host upon contact with an infectious individual and during Δt, and I_t _is the number of infectious hosts with which the i^th ^susceptible has contact.

The second transition probability is the probability that a pathogen in an infected host gives birth to C_min _new offspring, such that this host becomes infectious:(2)

where γ is the pathogen growth rate. Right after becoming infected, pathogen growth in a host is approximately exponential but it slows down as it reaches a maximum (C^Max^), at which it stops.

The last transition probability is the probability that C_min _pathogens are killed within the host:(3)

This equation follows from the dynamics between pathogens and immune factors, as observed in experimental studies [12,13]. Parameter μ represents the maximum number of pathogens killed for each unit of R^i^_t_, with R^i^_t _being a generic index related to the number, at time t, of the different types of immune factors specific for that pathogen. Because the main interest is on the number of pathogens, the complexity of the immune response is greatly simplified when R^i^_t _increases at a rate (h^i^) that is constant across time. The scaling parameter ρ^i ^varies from 0 to 1 and represents the extra investment in resistance of the i^th ^host with respect to μ. When C^i^_t _drops below C_min_, the infection is assumed to be cleared.

The Markov chain was simulated using the Gillespie algorithm [14], which essentially uses exponential waiting times between events. For all simulations, it was assumed that two individuals in the population were initially infected. Simulation steps were executed until t reaches T units of time (= one replicate) and repeated over 50 replicates. Each cycle took around 4 hours to complete, so the population size was limited at 30 individuals, which is the average size of most dairy herds in the Walloon region of Belgium.

### Individual performance

The performance of an infected host decreases proportionally to the number of pathogens (C^i^_t_) and to investments of the host in tolerance [15]:(4)

where P^i^_t _is the performance of the i^th ^host at time t, when it is infected with C^i^_t _pathogens, ω is the maximum amount of performance lost per pathogen (virulence). The parameter λ^i ^is a scaling parameter representing the extra investment in tolerance. If λ^i ^= 1, the host is completely tolerant and produces at a level identical to the one without infection. If λ^i ^= 0, the host is not tolerant to the deleterious effects of the pathogen.

Hosts invest part of their constitutive resources to resist or tolerate the pathogens and costs are assumed proportional to the investments in both types of defense. They are combined in an additive way:(5)

where P^Max ^is the totality of the resources available to the host to insure performance (e.g., production, reproduction, work) and to cope with an infection (resistance and tolerance). If no extra-investments are put in resistance and tolerance, all resources are allocated to insure the highest achievable level of performance in the absence of infection. Parameter c^i^_ρ _is the marginal cost of resistance and c^i^_λ _is the marginal cost of tolerance (in units of performance). Values for both costs are constrained such that the factor within brackets remains positive (ρ^i ^c^i^_ρ _+ λ^i ^c^i^_λ _≤ 1). A constraint was also set to insure P^i^_t _(equation 4) remains positive or null in totally non-tolerant individuals infected with K pathogens: ω ≤ P^Max^(1 - ρ^i ^c^i^_ρ_)/K.

**Figure 1:**
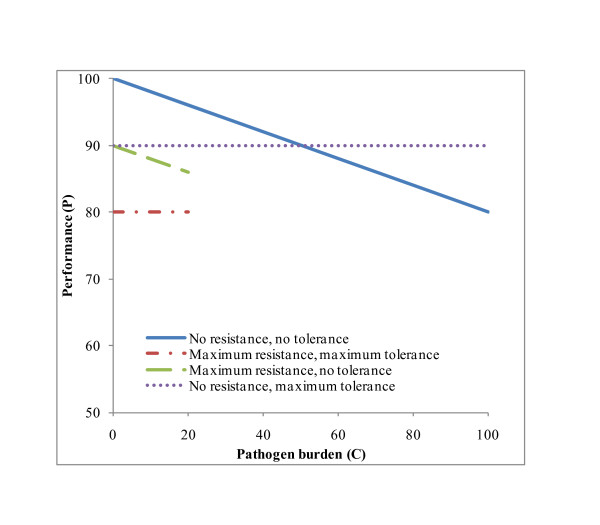
**Schematic representation of the impact of resource allocation on performance (P) and number of pathogens (C)**.

Typical patterns in performance as a function of number of pathogens are shown schematically in Figure 1 to illustrate the different ways resources can be allocated between resistance, tolerance and performance (costs are assumed equal for resistance and tolerance). Performances of hosts allocating none of the available resources to resistance and tolerance are the highest at the start of infection (P_t = 0 _= P^Max^) and decrease as C_t _increases. Numbers of pathogens remain below 20 among resistant hosts, and performances of tolerant hosts do not decline with increasing parasite burden.

### Effectiveness analysis

The most profitable strategy, i.e. the one that will insure the lowest number of infected animals or the highest performance of the population, or both, was identified by weighing the allowed extra investments in resistance, tolerance, or both, against the effectiveness of each of these alternatives.

Effectiveness was computed by comparing populations under the same infection process but in which animals invest ('yes' population) or not ('no' populations) in resistance, tolerance, or both. To do so, the number of infected hosts (I_t_) and the overall performance (P_t _= Σ_i = 1,N _P^i^_t_) were followed across time, and the area under the curves of P_t _(AUC_P_) and I_t _(AUC_I_) were obtained for t = 0 to T with the spline method of the procedure Expand of SAS^® ^[16]. Subsequently, the incremental effects (ΔE_I _and ΔE_P_) were computed as the difference between corresponding 'yes' and 'no' populations: ΔE_I _= AUC_I_^no ^- AUC_I_^yes^, and ΔE_P _= AUC_P_^yes ^- AUC_P_^no^. Then, the most effective alternative was identified as the one with the highest values for ΔE_I _and ΔE_P_.

Incremental effects were calculated for different sets of parameters (Table 1). Two transmission rates were considered, with β = 0.1 and β = 0.5, which correspond to a new infection per 10 and 2 effective contacts, respectively. The minimum number of pathogens was set to C_min _= 10 and the maximum to C^Max ^= 500. The growth rate (γ) was set at 0.5 new pathogens for each existing one and the value for μ was set to 0.25 or 1.0 to obtain killing rates equal to half or twice the pathogen growth rate. A convenient value of 100 was given to P^Max^, while virulence (ω) was set at 0.1 or 0.2 units of performance lost per pathogen present. Individual extra investments in resistance and tolerance were drawn from uniform distributions with different extreme values to have low (U[0, 0.5]), average (U[0, 1]), or high (U[0.9, 1]) levels of investments. Associated costs were drawn from uniform distributions within the allowable limits imposed by equations (4) and (5): U [0, 0.1], U [0.1, 0.2], and U [0.2, 0.5].

**Table 1:** Model parameters and their values

Symbol	Description	Values
P_t_^i^	Performance for animal i at time t	
C_t_^i^	Pathogen number in animal i at time t	
R_t_^i^	Immune response in animal i at time t	
I_t_	Number of infected animals in the population at time t	
P_t_	Population performance at time t	
ΔE_P_	Incremental effectiveness for P_t _over the T period	
ΔE_I_	Incremental effectiveness for I_t _over the T period	
N	Population size	30
T	Time duration	300
C_min_	Pathogen number necessary for infection	10
C^Max^	Maximum number of pathogens	500
P^Max^	Maximum performance	100
γ	Per-capita pathogen growth rate	0.5
β	Transmission rate	0.1; 0.5
μ	Maximum per-capita pathogen killing rate	0.25; 1.0
ω	Maximum performance loss per pathogen	0.1; 0.2
c^i^_ρ_	Marginal costs of resistance	U [0, 0.1]; U [0.1, 0.2]; U [0.2, 0.5]
c^i^_λ_	Marginal costs of tolerance	U [0, 0.1]; U [0.1, 0.2]; U [0.2, 0.5]
ρ^i^	Extra investment in resistance	U [0, 1]; U [0,0.5]; U [0.9, 1]
λ^i^	Extra investment in tolerance	U [0, 1]; U [0, 0.5]; U [0.9, 1]
h^i^	Rate of increase for the immune index	U [0, 0.001]; U [0, 0.01]; U [0, 0.1]

Finally, effects of low, average and high levels of extra-investments in resistance and tolerance on ΔE_I _and ΔE_P _were quantified using fixed linear models (proc GLM on SAS^® ^[16]) that also contained the effects of β, μ, and ω for the characteristics of the pathogen, the averages at the population level of h^i^, c^i^_ρ _and c^i^_λ _for the characteristics of the hosts, and all first-order interactions. The resulting least-squares estimates were used to identify epidemiological situations for which investments in tolerance, resistance or both were effective.

## Results

### Within-host pathogen dynamics

**Figure 2:**
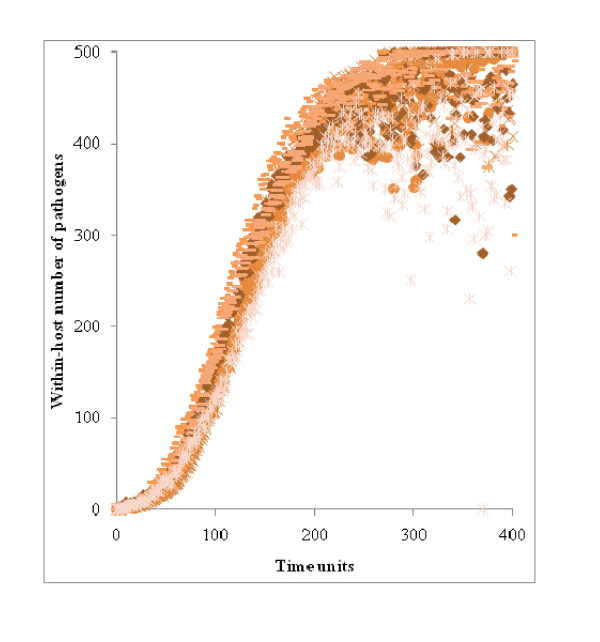
**Number of pathogens across time for 10 completely susceptible hosts**.

The number of pathogens within a host is shown in Figure 2 for 10 animals in a 'no' population with the following characteristics: β = 0.5; μ = 0.1; ω = 0.1; h^i ^~ U [0, 0.001], and ρ^i ^= λ^i ^= 0 for i = 1 to N. The duration and the number of pathogens generated were approximately the same for all animals because they depended on γ = 0.5 (equation 2). However, the stochastic nature of the simulation resulted in a cloud of points for each C^i^_t_. On average, C^Max ^was reached after 300 time units, so it was used as the upper limit for T because the Gillespie algorithm was slow to converge and because T = 300 insured the steady value C^Max ^was reached among completely non-resistant hosts.

**Figure 3:**
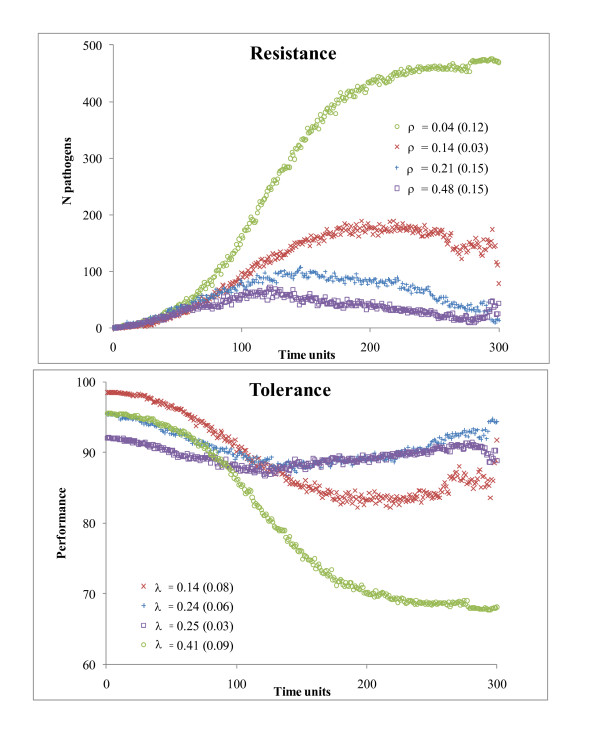
**Within-host dynamics for the number of pathogens and performances of four individuals with different levels of resistance (ρ) and tolerance (λ) **Associated costs are in parentheses.

In Figure 3, the dynamics in C^i^_t _and P^i^_t _are shown for four individuals with different investments and costs of resistance and tolerance, and for an infection with β = 0.5, γ = 0.5, ω = 0.2, h^i ^~ U [0, 0.1], and μ = 0.25. When both ρ^i ^and λ^i ^were high, C^i^_t _remained low and P^i^_t _did not change much across time (individuals □ or +). Conversely, when ρ^i ^was low, C^i^_t _increased up to its maximum and the associated individual performance decreased (individual ○ in Figure 3). Between these extremes, a wide range of different situations occurred. Initial performance (P_0_) varied according to the costs and extra investments in tolerance and resistance (equation 3).

### Between-host pathogen dynamics

**Figure 4:**
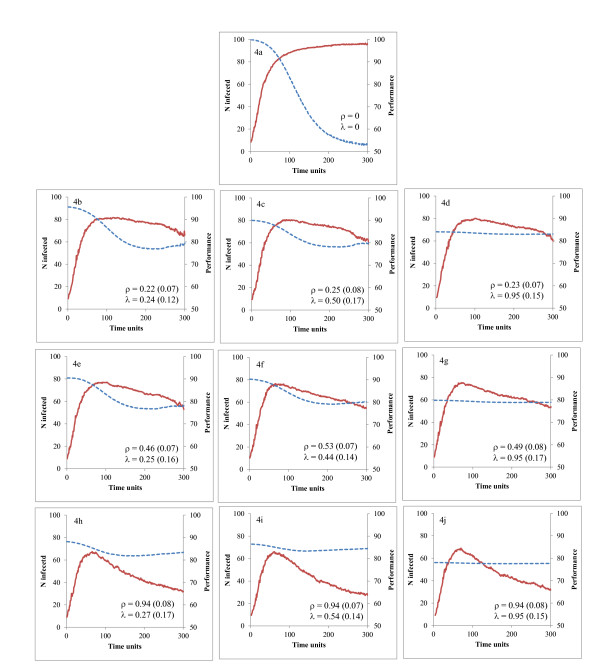
**Number of infected individuals (solid line) and overall performance (broken line) in populations with different average values for levels of resistance (ρ) and tolerance (λ), and for their associated costs (c**_**ρ **_**and c**_**λ **_**in parentheses) **The values are expressed as percentages of their maxima.

The number of infected hosts (I_t_) and the overall performance (P_t_) are given in Figure 4 as percentages of their maximum values (N and P^Max^, respectively) and for an infection with β = 0.5, γ = 0.5, ω = 0.1, and μ = 0.25. At T = 300, all individuals in the 'no' population (Figure 4a) were infected (with the exception of one) and the overall performance was close to 50%, which is the minimum expected from equation 5 when all animals have zero tolerance and are infected with C^Max ^pathogens.

When individuals invested more in resistance, only a fraction of the population got infected and AUC_I _was low. For example, AUC_I _decreased from 22,720 to 19,379 and 13,851 infected hosts in Figures 4b (ρ = 0.22), 4e (ρ = 0.46), and 4h (ρ = 0.94), respectively. When the average level of extra investments in tolerance was high (around 0.95), the impact of I_t _on P_t _was almost zero (Figures 4d,g and 4j). Otherwise, P_t _decreased as I_t _increased, especially for low levels of tolerance (Figures 4b,e and 4h). This should have translated in an increase in AUC_P _but, in this particular population, costs associated with tolerance were high (around 0.15) and initial performance was low. For example, P_0 _averaged 79.8 in Figure 4g (λ = 0.95; AUC_P _= 23,509) and 90.4 in Figure 4e (λ = 0.25; AUC_P _= 24,779).

### Effectiveness analyses

**Figure 5:**
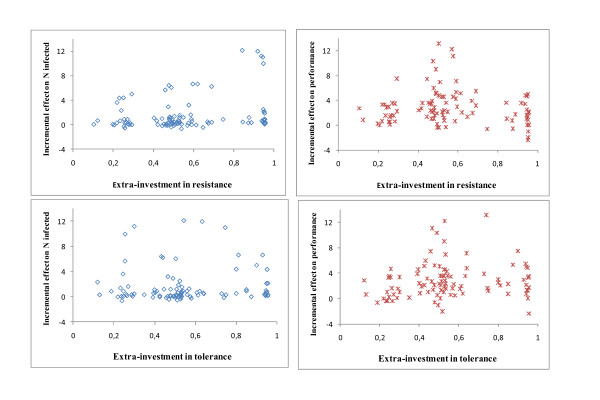
**Incremental effectiveness for performance (ΔE**_**P**_**) and number of infected individuals (ΔE**_**I**_**) for different investments in resistance (ρ) and tolerance (λ) and for various characteristics of the infection (Table 1) **.

Values of ΔE_P _and ΔE_I _obtained for each combination of the parameters of Table 1 are shown in relation to ρ and λ in Figure 5. Each dot corresponds to one specific combination of the parameter values. Effective combinations, those associated with both ΔE_P_>0 and ΔE_I_>0, represented 75.7% of all combinations. There was a tendency for ΔE_I _and ΔE_P _to increase with increasing values for ρ and λ, respectively. However, there were also combinations of parameters for which high values for ρ or λ were not effective, as revealed by the analysis of variance.

Results from the analysis of variance identified significant (p < 0.01) effects of ρ^i^, c_ρ_, h^i^, and μ on ΔE_I_, and of λ, c_λ_, β, and ω on ΔE_P_. All first-order interactions were non-significant (p > 0.10). Incremental effects are given in Tables 2 and 3 for selected combinations. Overall, ΔE_P _was greater for higher values of λ but, for moderately virulent (ω = 0.1) and slow spreading (β = 0.1) diseases, investments in tolerance were low or ineffective unless they incurred at low costs (Table 2). Investing in resistance (Table 3) was effective for infections that elicited moderate to high but not low (h^i ^~ U[0, 0.001]) immune responses in the hosts (unless levels of resistance were high).

**Table 2:** Incremental effectiveness of the performance of the population (ΔE_P_) associated to different investments in individual tolerance (λ^i^) and for selected values of c^i^_λ_, β and ω, as defined in Table 1

**λ**^**i **^**~ U[0,0.5]**				**λ**^**i **^**~ U[0.9,1]**			
**c**^**i**^_**λ**_	**β**	**ω**	**ΔE**_**P**_	**c**^**i**^_**λ**_	**β**	**ω**	**ΔE**_**P**_
0	0.1	0.1	1623	0	0.1	0.1	2974
U[0.2,0.5]	0.1	0.1	-628	U[0.2,0.5]	0.1	0.1	722
0	0.1	0.2	5635	0	0.1	0.2	6986
U[0.2,0.5]	0.1	0.2	3383	U[0.2,0.5]	0.1	0.2	4734
0	0.5	0.1	4198	0	0.5	0.1	5549
U[0.2,0.5]	0.5	0.1	1946	U[0.2,0.5]	0.5	0.1	3297
0	0.5	0.2	8210	0	0.5	0.2	9561
U[0.2,0.5]	0.5	0.2	5958	U[0.2,0.5]	0.5	0.2	7309

**Table 3:** Incremental effectiveness of the number of infected (ΔE_I_) associated to different investments in individual resistance (ρ^i^) and for selected values of c^i^_ρ_, μ and h^i^, as defined in Table 1

**ρ**^**i **^**~ U[0,0.5]**				**ρ**^**i **^**~ U[0.9,1]**			
**c**^**i**^_**ρ**_	**h**^**i**^	μ	**ΔE**_**I**_	**c**^**i**^_**ρ**_	**h**^**i**^	μ	**ΔE**_**I**_
U[0.2, 0.5]	U [0, 0.001]	0.25	-808	U[0.2, 0.5]	U [0, 0.001]	0.25	1509
0	U [0, 0.001]	0.25	-1215	0	U [0, 0.001]	0.25	1103
U[0.2, 0.5]	U [0, 0.001]	1	-127	U[0.2, 0.5]	U [0, 0.001]	1	2191
0	U [0, 0.001]	1	-533	0	U [0, 0.001]	1	1784
U[0.2, 0.5]	U [0, 0.1]	0.25	5607	U[0.2, 0.5]	U [0, 0.1]	0.25	7925
0	U [0, 0.1]	0.25	5201	0	U [0, 0.1]	0.25	7518
U[0.2, 0.5]	U [0, 0.1]	1	6289	U[0.2, 0.5]	U [0, 0.1]	1	8607
0	U [0, 0.1]	1	5882	0	U [0, 0.1]	1	8200

## Discussion

A general framework is proposed to provide insights into the effects of improved resistance and tolerance on the performance and size of an infected population. A clear distinction is made between effects of resistance on multiplication of the pathogen and effects of tolerance on damages induced by the pathogens. Hosts differ in the costs they incur to insure their particular levels of resistance and tolerance, and in the intensity of the response they mount against pathogens. Pathogens differ in their speed of spread between hosts, in virulence, and in the intensity of the response they elicit in the hosts. However, to be useful, the model must be validated and its limits and assumptions must be clarified, as will be discussed in the following, with examples mostly related to bovine mastitis.

### Validation of the model

Model validation usually takes the form of a comparison between model outputs and real data but this was not possible here because reliable field data are scarce, difficult to measure or imprecisely defined [17,18]. For example, estimates of costs associated with resistance and tolerance are limited in animals, in contrast to plants (see review by [19]). Tolerance has often been measured imprecisely as the overall ability to maintain fitness in the face of infection, irrespective of parasite burden. For example, cows infected with *E. coli *have been classified as moderate and severe responders according to milk production loss in the non-challenged quarters [20]. In this case, it is in reality a measure of the combined effects of resistance and tolerance [4]. It was also a deliberate choice to present a generic model because parameters values are different among disease and host populations, so model outputs for one specific disease may not apply to another disease. For example, transmission rates have been estimated at 0.20 to 1.50 per 1000 quarter-days at risk for *S. uberis *mastitis [21] but at 7 to 50 for *S. aureus *mastitis [22]. Similarly, killing rates have been estimated at 0.67 to 1.33 × 10^-8 ^mL/cell per min in milk of cows [23,24] and at 1.64 to 1.76 × 10^-8 ^mL/neutrophil per min in dermis of rats inoculated with *E. coli *[13]. Model outputs will also depend on the virulence of the invading pathogens (ω), as exemplified by the different amount of milk loss at the first occurrence of clinical mastitis depending on bacteria species [25], and on the type of performance (e.g., yield, quality of products, or capacity for work) considered.

As an alternative form of validation, the dynamics of C^i^_t _and P^i^_t _at the individual, and of I_t _and P_t _at the herd levels were evaluated. For instance, as expected, C^i^_t _was lowest in resistant and P^i^_t _was highest in tolerant hosts (Figure 3), P_t _remained stable across time when tolerance of the hosts was at its highest level, and I_t _decreased faster when resistance of hosts was at its highest level (Figure 4). Results from the analysis of variance also validated the model. The null value of ΔE_I _for h^i^~U [0, 0.001] was sensible because, at this low rate, pathogens cannot be killed, regardless of how much was invested in resistance. The fact that β did not affect ΔE_I _may also be explained by the same transmission in 'yes' and 'no' populations, so AUC_I_^yes ^was close to AUC_I_^no ^for any value of β. As a final example, ΔE_P _was higher for β = 0.5 than for β = 0.1 because only few animals got infected with β = 0.1, so improving tolerance of these few hosts was not beneficial at the population level.

### Limits and assumptions of the model

The strategy to build this model followed the current trend in epidemiology to begin with simple models and to add complexity only if the model fails to reproduce plausible epidemiological behaviors [26]. Several assumptions were made, some of which have been confirmed previously. One assumption was that available resources are partitioned between performance, resistance and tolerance. Indeed, experiences in poultry [27] and other species [28] have shown that individuals differ in their ability to allocate resources to their needs. This is also one of the factors evoked to explain the increased susceptibility of high yielding dairy cows to mastitis [29]. Lack of resources may lead to vicious cycles because hosts in poor condition are more susceptible to higher pathogen occurrence and infection intensity, which further weaken the condition of the host [30]. Another assumption is that investments in resistance and tolerance are linked through the constraint in equation 4 and this has been confirmed by [2], where a negative relationship was found between resistance and tolerance in rodent malaria.

Some assumptions of the model could also be relaxed with more complex equations that have been used in models examining the effects of mixed infection [21], infectious dose [31] and vaccination/treatment [32] on transmission dynamics. Resistance could vary as a function of exposure to disease [33]. Availability of external resource can vary across time, as in Doesch-Wilson et al. [34]. In the model used here, individual infectious contacts were assumed independent and at random but models with heterogeneous mixing [35] and that consider genetic susceptibility among relatives [36,37] may be more appropriate. The course of infection within hosts can also be modelled more accurately, in line with the characteristics of the disease under study. For example, models with increasing complexity have been proposed to describe the fate of mastitis-causing *E. coli *in infected cows [23,24]. Models for co-evolutionary mechanisms between host and pathogens should be considered [38] if the time scale is longer than the one used in this study.

Other assumptions may be difficult to verify. For such assumptions, a set of arbitrary standard values for the parameters and different forms for equations should be tested in so-called sensitivity analyses. For example, the amount of loss in performance was assumed directly associated with pathogen load, although the most dramatic changes may occur at low or subclinical levels of disease, with diminishing effects of each additional parasite [39].

### Effectiveness analyses

Two results from the effectiveness analyses are noteworthy, although they must be further evaluated in empirical studies. One is that the range of possible values of ΔE_P _and ΔE_I _for the different input parameters (Figure 5) is wide. This emphasizes the need to accurately model the infection process and its impact on the population before deciding on the most effective strategy. For example, increasing host tolerance is theoretically less effective for improving performance of populations infected with pathogens that cause minor rather than major mastitis. Indeed, pathogens causing minor mastitis are less virulent (ω) and less transmissible (β) than those causing major mastitis [40], so modest advantages of high tolerance would be offset by the associated costs. Likewise, selecting for better resistance to mastitis would be effective to restrict the size of a population epidemic if animals are infected with bacterial strains that are likely to be killed by neutrophils [41], i.e. μ>0 in equation 3.

Another noteworthy observation is that least-squares means for ΔE_P _were highest in highly tolerant populations, while ΔE_I _did not change between different tolerance levels. This suggests that selection for increased tolerance would be effective under commercial constraints. This is different from models applied to natural populations that predict an increase in the overall incidence of infection as the frequency of tolerant hosts increases [38]. In natural populations, tolerant hosts survive longer than non-tolerant ones, thus keeping the disease longer in the population and increasing the risk of exposure to disease. Here, the model is for an endemic disease in a population under commercial contraints, in which non-tolerant animals are kept even if they are sick (no natural death, no culling). Consequently, the risk of exposure to disease does not change, even if the pathogen population size (C) increases.

In general, little is known about tolerance mechanisms in animals but their study should provide a good foundation for insuring health over the long term. Indeed, in the long term, advantages of being tolerant should be greater than those associated with resistance. For example, in non-evolving pathogen populations, advantages of being resistant decrease in parallel with the decline in disease frequency, while the advantages of being tolerant are maintained, or even increase if disease frequency rises [42]. In evolving pathogen populations, improved host resistance will pressure pathogens to evolve better mechanisms to evade host defense processes, potentially resulting in cyclical co-evolutionary dynamics. In contrast, tolerance does not interact directly with the pathogen and should not induce selection for counter-adaptations, although elevated levels of tolerance may allow pathogens to be more virulent [43].

Practically, in bovine mastitis, it the degree of tolerance of an animal can be estimated by the amount of milk loss per bacteria present in the quarter (CFU) using a model adapted from that proposed by [2] for inbred strains of laboratory mice:

where y_ij_^t ^is the milk yield at time t of the i^th ^cow (yield corrected for fixed and non-genetic random effects estimated from the genetic evaluation model) infected with I_ij_^t^, i.e. the bacterial load for bacterial species j; b_j _is the average tolerance against bacteria of strain j; B_ij _describes individual random deviations from the average tolerance with B_ij _~ IID N(0, σ²_b_); and e_ij_^t ^are residuals with e_ij_^t ^~ N(0, V_e_), where V_e _accounts for the non-independence between repeated e_ij_^t^. Such information could be collected from quarters of experimentally infected cows, as was done in the study of [44].

## Conclusions

In summary, this paper presents a novel epidemic model to explore the effects of tolerance and resistance on performance and disease spread in a population. Although more research is necessary to validate the model and more empirical studies are needed to obtain values for the input parameters, the analytic approach can be used to find optimal strategies of disease control in commercial populations.

## Competing interests

The authors declare that they have no competing interests.
